# Life After the HSIF: Lessons From Diffusion of Innovation for Sustaining the Impact of Embeddedness: Comment on "Early Career Outcomes of Embedded Research Fellows: An Analysis of the Health System Impact Fellowship Program"

**DOI:** 10.34172/ijhpm.8663

**Published:** 2024-11-13

**Authors:** Mark Embrett, Meaghan Sim

**Affiliations:** Implementation Scient Team, Health Innovation Hub, Nova Scotia Health, Halifax, NS, Canada.

**Keywords:** Diffusion of Innovations, Health System, Embedded Science, Sustainability, Knowledge-to-Action

## Abstract

Kasaai and colleagues examine the career outcomes of alumni from the Canadian Institutes of Health Research’s (CIHR’s) Health System Impact Fellowship (HSIF), which embeds emerging scholars in health system organizations. The study of the 2017-2019 cohort shows all alumni are employed, with 92% working in Canada, and highlights their presence in academia, public service, healthcare, and private industry. Notably, 37% hold "hybrid" roles, blending academic and other sector work. While the fellowship effectively prepares fellows for impactful careers, the prevalence of hybrid roles raises concerns about sustaining academic partnerships post-fellowship. This commentary explores risks to embedded scholars, such as decentralization, competing innovations, and limited ongoing training, using the Diffusion of Innovations framework. It suggests strategies like strengthening network connectivity, focusing on high-impact innovations, increasing organizational embeddedness, and maintaining adaptability to ensure the long-term success of embedded scholars and the integration of evidence-based innovations in health systems.

## Embeddedness Beyond the Health System Impact Fellowship: Sustaining

 The article by Kasaai and colleagues focuses on exploring the career trajectories of alumni of the Canadian Institutes of Health Research’s (CIHR’s) Health System Impact Fellowship (HSIF) – a strategic program of CIHR’s Institute of Health Services and Policy Research designed to support career readiness for emerging scholars by situating them within health system organizations, as well as advancing capacity for learning health systems. As fellows from the original HSIF cohort, and currently holding embedded scientific roles within a large provincial health authority,^1^ we are enthused by the findings of this study and the opportunity to reflect on its implications for supporting current and future fellows and a learning health system culture across Canada and beyond.

 Kasaai et al analyzed HSIF alumni’s (2017-2019 cohort, n = 87) employment through publicly available data on employment status, supplemented with information from a post-fellowship survey. The results showed that all alumni were currently employed, with 92% working in Canada.^2^ The alumni were employed across various sectors, including academic (37%), public (29%), healthcare delivery (17%), and private (14%). Additionally, 37% of alumni held “hybrid” roles with affiliations in academia and another sector. The most common positions held by alumni were either senior scientist, professorships, and director/manager roles. The study found that the employment outcomes of the alumni were generally consistent with their career aspirations reported at the start of the fellowship program. Overall, the findings suggest that the HSIF program has been successful in one of its goals of preparing fellows for impactful careers within and beyond health systems.

 In this response, aimed at fellows, health organizations and academic institutions interested in sustainable impact through the HSIF, we review the findings from Kasaai and colleagues’ study and identify a potential challenge within the HSIF alumni’s career path meriting further exploration regarding the “hybrid” position of which nearly a third of the surveyed alumni occupy. The term “hybrid” departs from the embedded scholar definition of someone deliberately working in both academic and health system roles.^3^ Within this commentary, the embedded role is presented as a scientist working in a health system, without academic (or other health system actor) affiliation. This is a change from the original definitions used for HSIF as embedded scientists and should not be overlooked. During the fellowship (as part of the embedded role) there is a requirement for health organizations and academic institutions to form a partnership and work collaboratively to support the health system impact (HSI) fellow and their goals. A substantial part of the fellowship application evaluation criteria rests in both parties articulating how this partnership will be achieved.^4^ However, following completion of the fellowship, even if the fellow remains with the same health system organization, this requirement ceases, and the academic institution is no longer required to participate in mentorship or collaborate with the fellow alumnus – even if the synergies of such networks are recognized as important accelerators to achieve learning health system goals.^5^ The change in this role and its implications for continued learning health system capacity requires further examination.

 HSI fellows have described themselves in their roles as catalysts for change, knowledge brokers, and collaboration facilitators within the health system.^6^ Their role is designed to increase the impact of research evidence by placing scientists within health system organizations, where they work closely with decision-makers and practitioners to promote evidence-informed practices, foster transformation, and encourage interdisciplinary collaboration. This real-time engagement ensures that scholars are not only co-producing relevant knowledge but also influencing how evidence is integrated into decision-making processes.

 Embedded scholars represent an innovation in the health system because they address a persistent gap between research production and practical implementation. Traditional knowledge transfer methods—such as publishing research or presenting findings at conferences—often struggle to meet the immediate, context-specific needs of health systems. By embedding researchers directly within organizations, this model ensures continuous interaction between science and practice, making research outputs more relevant, timely, and actionable. Scholars bring not only scientific rigor but also adaptability, embedding themselves within the operational context to create practical solutions to real-world challenges.

 The degree of embeddedness is defined by how deeply the scholar participates in core activities of the health system, such as problem-solving alongside practitioners and decision-makers, adapting evidence to meet emerging challenges, and contributing to the organization’s strategic goals. Through these “embedded activities,” scholars become integral agents of change, promoting knowledge co-production and effective evidence translation.

 This embedded model is innovative because it transforms scholars from external consultants into internal collaborators who may directly influence organizational decision-making and culture. Their proximity to knowledge users allows them to align research outputs with immediate needs, combining academic findings with practical insights to improve health services and policies. Embedded scholars also bridge disciplinary silos by fostering collaboration across departments, blending research evidence with grey literature, and co-creating interventions that are both evidence-based and feasible to implement.

 By engaging across multiple sectors and collaborating with diverse partners, scholars strengthen the system’s capacity for innovation. Furthermore, they emphasize relationship-building and trust, which are critical for sustaining effective multi-sectoral partnerships and driving long-term organizational improvement. Their ability to network, integrate diverse viewpoints, and adapt to evolving needs makes embedded scholars a valuable innovation in promoting dynamic, evidence-informed change within the health system. They also work with partners across various sectors to address critical health system challenges through integrated knowledge translation.^6^ To be successful, all these embedded activities require leadership buy-in, clear role expectations, and institutional support mechanisms like cross-sector mentorship and governance engagement.

 The embedded role is crucial in ensuring that the latest research findings are translated into practice, thereby improving patient outcomes and the efficiency of health services. However, there is a risk of the organization itself as well as the scientist to revert to less-than-ideal standards of science and its methods. This risk grows without the scientists’ academic anchor that helps sustain scientific integrity, supports professional development and academic networks, and remain abreast of new approaches and training in their chosen subject matter – or in the health services and policy research field more broadly.

 To better understand this possibility, the concept of Diffusion of Innovations—how new innovations spread within and between organizations (ie, the new embedded role)—can be used to examine the risk to the sustained impact of the role of embedded scholars (as described above). This commentary explores the potential threats to embedded scholars exposed by the Diffusion of Innovations and suggests strategies to mitigate these risks and argues the need for a continued anchor to academia for impactful health system and policy research.

###  The Diffusion of Innovations Framework

 As described, embedded scientists are an innovation in health systems, therefore, along with our own perspectives, we apply a theory of innovation to the role,^7^ which suggests there is a risk that the fellow’s embedded role will adjust to the norm of the health organization, and there will be no more training or skilling up of core competencies. The Diffusion of Innovations framework describes how new ideas, practices, or products spread within a social system over time.^8^ The framework identifies five key stages in the adoption process: knowledge, persuasion, decision, implementation, and confirmation. Factors influencing diffusion include the perceived attributes of the innovation (relative advantage, compatibility, complexity, trialability, and observability), communication channels, time, and the social system. Greenhalgh and colleagues expanded the application of Rogers’ original framework to conceptualize the Diffusion of Innovations within health service organizations.^9^

###  Risks to Embedded Scholars

 There are several threats to the impact of HSI fellows, that we perceive as possible after serration from academia. These threats include:

 Decentralization and fragmentation: The diffusion process can lead to decentralization and fragmentation within health systems, which may dilute the influence of embedded scholars. As innovations spread across different regions and institutions, research efforts of the embedded scientists are spread out, reducing the consistency and coherence of implementation efforts. This phenomenon is observed in studies examining the diffusion of competing innovations in healthcare networks, where disparities in adoption rates are linked to network clustering and geographical variations.^10^

 Competing innovations: The presence of multiple, competing innovations can create a challenging environment for embedded scholars. Although competing innovations and priority setting are a challenge for all healthcare organizations^11^ embedded scholars face distinct difficulties in this context as it relates to timely adoption of evidence-informed innovation intended to improve health system outcomes. Their role includes considering evidence from various perspectives which may result in divided attention while having limited resources, making it more difficult to prioritize and demonstrate the impact of their work within the time constraints which may lead to slower and less effective implementation. This competition, along with balancing of perspectives, can hinder the ability of embedded scholars, who were focused on fewer projects during the fellowship, to demonstrate the impact of their work and secure continued support.^12^

 Variability in organizational support: Organizational support for embedded scholars can vary significantly, affecting their ability to influence health systems. High embeddedness, where scholars are deeply integrated within the organization, can facilitate better alignment with strategic goals and more effective implementation. Conversely, low embeddedness can limit their access to resources and decision-making processes, reducing their impact.^6^

 Evolving priorities and contexts: Health systems are dynamic environments. As new challenges and opportunities arise, the focus of embedded scholars may need to shift, potentially leading to the abandonment or modification of ongoing initiatives. This flexibility, while necessary, can undermine the long-term impact of embedded research if not managed carefully.^13^

 Lack of ongoing training or professional development: There is a necessity for additional training and capacity development to support the career progression of early career embedded scientists. Areas such as data science, informatics, and other relevant skills were identified as crucial for enhancing their impact in healthcare settings.^3^ Health systems are well known for their absence of providing ongoing training or professional development opportunities.^14^ Opportunities for ongoing training and professional development may also impact on mid- and later-career stage embedded scientists who need to stay abreast of the impact of Health System Policy Research in the digital age.^15,16^

###  Strategies to Mitigate Risks

 Despite these threats, there are opportunities to mitigate them, if implemented and sustained. Fundamental to each mitigation strategy is the need for healthcare organizations, universities, and policy-makers to collaborate closely to define clear roles, establish expectations, and provide sustained support. Position descriptions should highlight the importance of scholars having structured access to decision-making platforms, opportunities for continuous professional development, and guidance through cross-sector mentorship. Strong leadership backing is crucial to fostering an environment where scholars can effectively shape strategic priorities and adapt to changing contexts with confidence. Mitigation strategies may include:

 Strengthening network connectivity: Enhancing connectivity within and between health system networks can help mitigate the risks of decentralization and fragmentation. By fostering strong, collaborative relationships, embedded scholars can ensure more uniform adoption and implementation of innovations across different regions and institutions. This approach emphasizes the importance of centralized diffusion systems that facilitate coordinated efforts.^17^

 Prioritizing high-impact innovations: To address the challenges posed by competing innovations, embedded scholars should prioritize high-impact innovations that align with organizational goals and demonstrate clear benefits. This prioritization requires rigorous evaluation and strategic decision-making to focus efforts on innovations with the greatest potential to improve patient outcomes and system efficiency.^18^

 Enhancing organizational embeddedness: Increasing the degree of embeddedness can enhance the impact of embedded scholars. This involves ensuring scholars are integrated more deeply into organizational structures and processes by continuously engaging with practitioners and decision-makers relevant to their role. This includes ensuring they have a voice in strategic planning and decision-making. High embeddedness, considered having an established role that includes routine involvement of described “embedded activities,” can lead to better alignment with organizational goals and more effective implementation of evidence-based innovations.^19^

 Adaptation and flexibility: Embedded scholars must remain adaptable and responsive to changing priorities and contexts within health systems. This requires ongoing communication with stakeholders, continuous evaluation of implementation efforts, and the ability to pivot when necessary. By maintaining flexibility, embedded scholars can sustain their impact and relevance in dynamic environments.^20^

###  Scale, Spread and Sustain

 While the spread of new ideas and practices can drive significant improvements in patient care, it also poses risks to the sustained impact of embedded research. By strengthening network connectivity, prioritizing high-impact innovations, enhancing organizational embeddedness, and maintaining adaptability, embedded scholars can navigate these challenges and continue to contribute effectively to the transformation of health systems. Their role remains crucial in ensuring that evidence-based innovations are implemented in a manner that maximizes benefits for patients and health systems alike. By following our suggestions, embedded scientists may navigate the complexities of studying healthcare innovation, address barriers to spread, sustainability, and scale-up, and work towards achieving meaningful and sustainable transformation within learning health systems.

## Ethical issues

 Not applicable.

## Conflicts of interest

 Authors declare that they have no conflicts of interest.
